# Impairment of circulating endothelial progenitors in Down syndrome

**DOI:** 10.1186/1755-8794-3-40

**Published:** 2010-09-13

**Authors:** Valerio Costa, Linda Sommese, Amelia Casamassimi, Roberta Colicchio, Claudia Angelini, Valentina Marchesano, Lara Milone, Bartolomeo Farzati, Alfonso Giovane, Carmela Fiorito, Monica Rienzo, Marco Picardi, Bice Avallone, Massimiliano Marco Corsi, Berardo Sarubbi, Raffaele Calabrò, Paola Salvatore, Alfredo Ciccodicola, Claudio Napoli

**Affiliations:** 1 Institute of Genetics and Biophysics ''A. Buzzati-Traverso'', IGB-CNR, Naples, Italy; 2 Section of Microbiology, Department of Experimental Medicine, 1st School of Medicine, Second University of Naples, Naples, Italy; 3 Department of General Pathology and Excellence Research Center on Cardiovascular Diseases, 1st School of Medicine, Second University of Naples, Naples, Italy; 4 IRCCS Fondazione SDN, Naples, Italy; 5 Istituto per le Applicazioni del Calcolo, "Mauro Picone", CNR, Naples, Italy; 6 Department of Biological Science, University of Naples "Federico II", Naples, Italy; 7 Department of Biochemistry and Biophysics, Second University of Naples, Naples, Italy; 8 IRCCS Multimedica, Milan, Italy; 9 Department of Biochemistry and Medical Biotechnology, University of Naples "Federico II", Naples, Italy; 10 Institute of General Pathology, Section of Clinical Pathology, Faculty of Medicine, University of Milan, Milan, Italy; 11 Cardiology Department of Second University of Naples, "Monaldi Hospital", Naples, Italy; 12 Department of Cellular and Molecular Biology and Pathology "L. Califano" and School of Biotechnological Sciences, University of Naples "Federico II", Naples, Italy

## Abstract

**Background:**

Pathological angiogenesis represents a critical issue in the progression of many diseases. Down syndrome is postulated to be a systemic anti-angiogenesis disease model, possibly due to increased expression of anti-angiogenic regulators on chromosome 21. The aim of our study was to elucidate some features of circulating endothelial progenitor cells in the context of this syndrome.

**Methods:**

Circulating endothelial progenitors of Down syndrome affected individuals were isolated, *in vitro *cultured and analyzed by confocal and transmission electron microscopy. ELISA was performed to measure SDF-1α plasma levels in Down syndrome and euploid individuals. Moreover, qRT-PCR was used to quantify expression levels of *CXCL12 *gene and of its receptor in progenitor cells. The functional impairment of Down progenitors was evaluated through their susceptibility to hydroperoxide-induced oxidative stress with BODIPY assay and the major vulnerability to the infection with human pathogens. The differential expression of crucial genes in Down progenitor cells was evaluated by microarray analysis.

**Results:**

We detected a marked decrease of progenitors' number in young Down individuals compared to euploid, cell size increase and some major detrimental morphological changes. Moreover, Down syndrome patients also exhibited decreased SDF-1α plasma levels and their progenitors had a reduced expression of SDF-1α encoding gene and of its membrane receptor. We further demonstrated that their progenitor cells are more susceptible to hydroperoxide-induced oxidative stress and infection with Bartonella henselae. Further, we observed that most of the differentially expressed genes belong to angiogenesis, immune response and inflammation pathways, and that infected progenitors with trisomy 21 have a more pronounced perturbation of immune response genes than infected euploid cells.

**Conclusions:**

Our data provide evidences for a reduced number and altered morphology of endothelial progenitor cells in Down syndrome, also showing the higher susceptibility to oxidative stress and to pathogen infection compared to euploid cells, thereby confirming the angiogenesis and immune response deficit observed in Down syndrome individuals.

## Background

Down syndrome (DS) is a complex disorder caused by trisomy of the entire or a critical portion of chromosome 21 (HSA21); it represents the most frequent genetic cause of mental retardation, with a frequency of about 1/1000 new-borns, and is associated with a huge number of congenital heart defects [1]. DS individuals have also an increased risk of early-onset Alzheimer disease [1]. Immunological and autoimmune disturbances, with high rates of infections and malignancies, are recurrent phenomena in DS pathogenesis [2], and infections still represent major cause of death in DS [3,4]. Despite the increased risk of leukaemia, DS patients have a low incidence to develop solid tumors [5,6], and a reduced incidence of diabetic retinopathy, suggesting, at least in part, a common angiogenesis' suppression [5,7]. Impaired endothelial function at a young age, possibly due to increased oxidative stress and yet unknown mechanisms, is a common DS feature [8].

DS phenotype results from a dosage imbalance of HSA21 genes, although expression analyses have reported conflicting results [9,10]. The over-expression of chromosome 21 genes greatly varies across the trisomic tissues [11,12], and analyzing specific cell type/tissue, in easy-accessible and non-invasive manner, may be more productive [13,14].

Growing interest is emerging on circulating endothelial progenitor cells (EPCs) and their pivotal role in the maintenance of endothelium integrity, repair after injury and postnatal neovascularization [15-17]. Many studies are providing encouraging insights into the use of EPCs in the clinical setting [18,19]. Indeed, accumulating evidences indicate a reduced availability, and/or impaired EPC function, in cardiovascular and metabolic diseases [17,20,21]. EPCs number was recently shown to be impaired in DS fetuses and children [22,23] and CD34+ haematopoietic progenitors exhibited a marked growth decrease in Ts65Dn - a DS mouse model - accounting, at least in part, for DS vascular anomalies and defective immune response to pathogens [24].

Bacterial toxins may trigger pathogenic events through the over-production of cytokines and chemokines, leading to the alteration of endothelial function and capillary leakage [25]. Particularly, we recently demonstrated [26] that *Bartonella henselae*, a gram-negative intracellular bacteria responsible of vasoproliferative disorders in immunocompromised individuals [27,28], adheres to and invades EPCs.

The present study was designed to pursue the molecular mechanisms contributing to immune, vascular and haematopoietic defective DS phenotypes, by investigating the number and functions of DS EPCs compared to euploid cells, also focusing on bioinformatics analysis of differentially expressed genes. Moreover, by using the previously described *B. henselae *model, we investigated the susceptibility of DS progenitors to this pathogen infection, also performing a detailed analysis of deregulated genes after Bartonella infection, with particular attention to angiogenesis and immune response pathways.

## Methods

### Subjects

DS and euploid donors were recruited at the Institute of General Pathology, Section of Clinical Pathology, Faculty of Medicine, University of Milan, and at the Second University of Naples, and an approval statement was obtained by the ethics' review boards of both Institutions. Informed consent was obtained from all persons involved in all clinical investigation of this study according to the principles expressed in the Declaration of Helsinki.

All subjects recruited for EPC isolation were free of infection, and no individual was taking any medication known to affect immune system/response. DS and euploid individuals were 65% males and 35% females as gender and 28 ± 9 as mean age. The experiments, where not specified, were performed on at least six DS and age-matched euploid individuals.

Plasma samples were obtained from 50 DS individuals and 30 age matched euploids subdivided into three age subgroups (young 0-20 y.o.; adult 21-40 y.o.; old 41-60 y.o.) as described elsewhere [29].

### EPC Isolation

EPCs were isolated from non-institutionalized individuals with DS and age-matched euploid donors.

EPCs were isolated as previously described [30]. Briefly, PBMCs were isolated by density gradient centrifugation of peripheral blood samples on Histopaque-1077 (Sigma). Cells were washed twice with PBS and counted. PBMCs were plated on culture dishes pre-coated with gelatin and fibronectin and maintained in endothelial basal medium-2 (EBM2; Cell Systems). Cells were cultured at 37°C with 5% CO2 in a humidified atmosphere. After four days, non-adherent cells were removed and adherent cells were used for further analyses.

### Bacterial strains and growth conditions

The *B. henselae *strain ATCC 49882 (LGC Promochem) was grown on Columbia agar supplemented with 5% defibrinated sheep blood (Oxoid) in a humidified atmosphere at 37°C and 5% CO2. For production of bacterial stock suspensions, bacteria were harvested after 7 days of culture until they reached the mid-exponential phase of growth (109 bacteria/ml), resuspended in Tryptone Soya Broth USP (Oxoid) containing 10% glycerol, and stored at -80°C. The number of viable bacteria in the frozen stocks was determined as previously described [26].

### B. henselae infection

For infection, Bartonella stock solutions were thawed, washed and suspended in antibiotic-free cell culture medium, and sedimented onto cultured EPCs at different multiplicity of infection (MOI) of 50, 100, 250, 500 and 1000 [26]. The MOI for infections was confirmed by plating serial dilutions of the infection inoculum. Assays were performed three times in triplicate.

### Confocal immunofluorescence microscopy

EPCs were dual stained with Dil-Ac-LDL and lectin from *Ulex europaeus *and counted both by fluorescence microscopy and flow cytometry as previously described [26,30]. Images were obtained by Zeiss LSM 510 with plan-apochromat X 63 (NA 1.4) oil immersion objective. EPCs images were used to measure cell size with ImageJ (http://rsb.info.nih.gov/ij/).

### SDF-1α plasma levels

Commercially available SDF-1α ELISA kit (Quantikine, R&D Systems) was used to determine plasma SDF-1α levels. Tests were carried out at RT on freshly thawed plasma samples of 50 DS individuals and 30 age matched euploids subdivided into three age subgroups (young 0-20 y.o.; adult 21-40 y.o.; old 41-60 y.o.) [29]. Concentration was determined by comparison with a standard curve, following manufacturer's instruction.

### Transmission electron microscopy

After a short incubation with Trypsin/EDTA, DS and euploid EPCs, both infected and uninfected with *B. henselae*, were harvested, centrifuged and washed in PBS. After centrifugation at a speed of 400 g for 7 min, cells were fixed in 4% glutaraldehyde as described [31]. Postfixation, dehydratation of specimen, semithin (2 μm) and ultrathin (80 nm) sections were performed as previously described [26]. Semithin sections were analysed with a light microscope (Polivar Reichert-Jung). Ultrathin sections were examined with Leo 912 AB transmission electron microscope operating at 80 kV.

### C11-BODIPY581/591 fluorescence

Oxidation-sensitive fluorescent probe, C11-BODIPY581/591 (C11-BO, Invitrogen), was loaded (2 μM final concentration) into the cells 30 min. before oxidative treatment. The samples were aliquoted in triplicate wells of a 24-well microplate, and fluorescence was determined with confocal laser microscopy at different times (0, 1, and 6 hours) from oxidant treatment. Between times, plates were maintained at 37°C in 5% CO2. To determine red fluorescence each microplate was excited at 543 nm (emission at 590 nm); for green fluorescence, microplates were excited at 488 nm (emission at 526 nm). Blank wells were also evaluated as well as C11-BO alone.

### Microarray analysis and quantitative RT-PCR

Total RNA (10 μg) was isolated as previously described [32] [Additional file 1: Supplementary Methods]. A pool of three samples (a total 15 μg of cRNA) was used for each hybridization - 2 pools of three infected and three uninfected euploid and DS - on the Affymetrix U133 2.0 probe array cartridge as described elsewhere [26]. Microarray data were submitted to Array Express (http://www.ebi.ac.uk/arrayexpress; provisional accession number E-MTAB-312). Results were validated by qRT-PCR and semi-qRT-PCR, performed as described [32], using primer pairs listed in [Additional file 2: Supplemental Table S1].

### In silico significant pathway identification

Analysis of over-represented genes was performed using the Database for Annotation, Visualization, and Integrated Discovery (DAVID) [33,34] and the PANTHER (Protein ANalysis THrough Evolutionary Relationships) Classification System [35 [Additional file 1: Supplementary Methods].

### Statistical analysis

EPCs number, cell size differences, SDF-1α plasma levels, fluorescence intensity of C11-BO and qRT-PCRs data were reported as mean values, and results analysed by paired Student t test. *P *value < 0.05 was considered statistically significant [Additional file 1: Supplementary Methods].

## Results

### EPC number and phenotype

**Figure 1:**
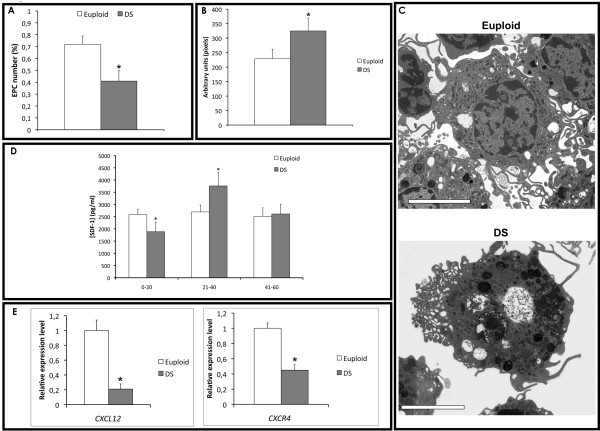
**EPC number, mobilization and morphology in DS**. A) EPCs number in euploids and DS determided as Dil-Ac-LDL/Lectin double positive cells (*P < .0001). B) EPC size measured by confocal microscopy and ImageJ (*P < .0001). C) Morphological characterization by TEM of EPCs isolated from euploids and DS. Scale bar: 5 ìm. D) SDF-1α plasma levels in DS and euploids (*P < .0001). E) Relative expression levels of CXCL12 and CXCR4 genes in euploids and DS measured by qRT-PCR (*P < 0.05).

We established that the number of EPCs isolated from peripheral blood of young and adult (mean age 28 ± 9) DS was significantly lower than age-matched euploid individuals (*P *< 0.0001 vs euploid EPCs; see Figure 1A). Phase contrast fluorescent microscopy [Additional file 3: Supplemental Figure S1A] and FACS analysis (data not shown) were used to identify double-positive cells for Dil-Ac-LDL and lectin [26,30]. By confocal microscopy and TEM we also observed early signs of cytoplasmatic disruption in DS progenitors, and cell size increase compared to euploid. In particular, by using ImageJ, we measured the cell size of DS EPCs, showing a significant increase compared to euploid cells (Figures 1B and Additional file 3: Supplemental Figure S1B]). We also measured cell cycle progression of DS progenitors vs euploid cells and we did not find any significant difference [Additional file 3: Supplemental Figure S1C]. Moreover, the ultrastructural examination revealed an increased number of phagolysosomes and vacuolization of DS progenitors compared to euploid cells (Figure 1C).

### SDF-1α plasma levels

EPC number is known to correlate to chemokines' plasma levels, such as SDF-1α (stromal derived factor-1α). Thus, we first measured by ELISA its plasma levels in peripheral blood of 30 euploid and 50 DS individuals, collected in three age subgroups [29]. Then, comparing mean SDF-1α values we found a significant decrease of SDF-1α plasma levels in young and adults DS compared to age-matched euploid individuals (*P *= 0.02) (Figure 1D).

Moreover, we measured by quantitative RT-PCR the expression of *SDF-1α *encoding gene, *CXCL12*, and of its membrane receptor *CXCR4*. A significant decrease in the expression of *CXCL12 *(5-fold; *P *< 0.05) and *CXCR4 *(2-fold; *P *< 0.05) was observed in DS endothelial progenitors compared to euploid cells (Figure 1E).

### *B. henselae *infection of endothelial progenitors

**Figure 2:**
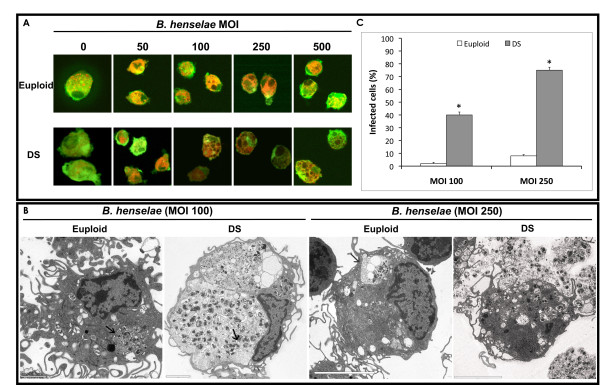
***B. henselae *infection**. EPCs isolated from DS and euploids were infected at different MOI of Bartonella as indicated. A) Confocal images of uninfected and infected EPCs at indicated MOI. Cells were stained with Dil-Ac-LDL (red) and lectin (green). B) TEM images of DS and euploid EPCs infected at the MOI of 100 and 250. Invasomes are highlighted by arrows. Scale bars are 5 ìm, 2 ìm, 5 ìm and 5 ìm, respectively. C) Bar graph representation of DS and euploid infected cells at different MOI.

EPCs isolated from young euploid and DS individuals were infected after 3 days of culture with *B. henselae *at different MOI as described elsewhere [26] (Figure 2A). TEM examination confirmed that *B. henselae *is internalized by endothelial cells as bacterial aggregates within invasomes or as single bacteria by protrusions of the cells. Interestingly, the EPC number was dramatically impaired in both DS and euploid after bacteria internalization [Additional file 3: Supplemental Figure S1D]. In contrast, by confocal microscopy at high magnifications (x630), a more detrimental effect was observed in infected DS progenitors, showing some morphological major differences compared to euploid EPCs when the same non-lethal MOI of Bartonella was used (Figure 2A). Ultrastructural analysis revealed that infected DS progenitors have increased intracellular accumulation of bacteria, forming invasomes, compared to euploid cells infected at the same MOI of 100 (Figure 2B). Cytoplasmic protrusions of cell membranes were also observed in both samples following adherence of the bacteria to the host cells. Moreover, after infection at higher MOI (250), DS progenitors showed larger invasomes, also displaying, in some cases, invasome and cell membrane rupture with subsequent bacteria outflow (Figure 2B). In contrast, infected euploid cells showed significant lower number of invasomes. The number of infected DS cells was estimated to be significantly higher compared to euploid cells at both MOI used (Figure 2C). We did not use infection at MOI ≥500 of *B. henselae *since they were not compatible with DS progenitors' survival.

### Oxidative stress in DS progenitors

**Figure 3:**
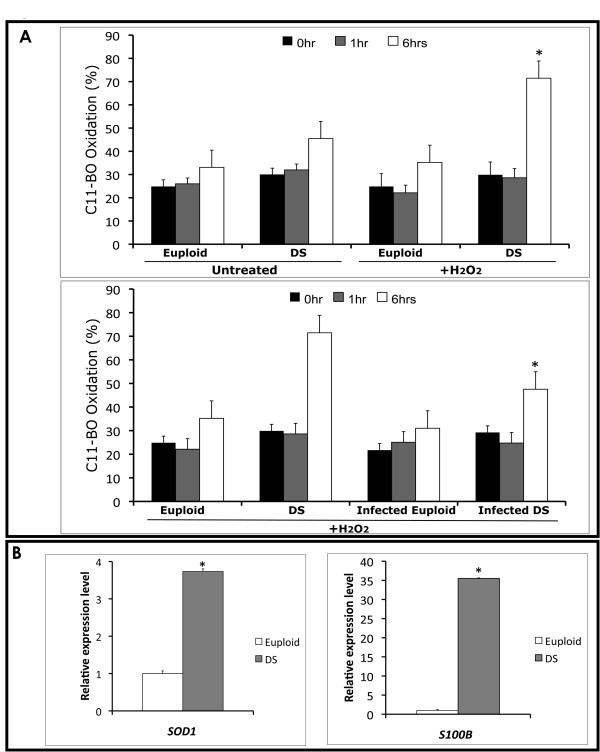
**Oxidative stress of EPCs**. A) Bar graph representation of C11-BO oxidation at different times (0, 1 and 6 hours) in DS and euploid EPCs in presence of hydroperoxide (200 ìM). Upper panel, C11-BO oxidation in hydroperoxide-treated euploid EPCs compared to DS (*P < 0.01). Lower panel, Bartonella-infected euploid and DS progenitors treated and untreated with hydroperoxide (*P < 0.01). B) *SOD1 *and *S100B *gene expression in euploid and DS EPCs by qRT-PCR. Data are shown as relative expression levels (Euploid EPC expression = 1).

To determine oxidant activities in living cells, membrane lipid peroxidation (LP) of isolated EPCs was measured by using C11-BO, a fatty acid analogue. We cultured both DS and euploid EPCs in the presence of a fixed concentration of hydroperoxide (200 nM) [36], and performed a time-course (0, 1 and 6 hours) experiment measuring the fluorescence emission by confocal microscopy. Particularly, in 6-hours treated DS samples, we observed a significant shift (Figure 3A upper panel) in the fluorescence emission from red towards green (590 nm to 520 nm) compared to untreated DS EPCs (*P *< 0.01). This shift was not observed in euploid hydroperoxide-treated EPCs vs untreated, as already described [36].

To evaluate the protective effects of *B. henselae*, known to induce - albeit at low MOI (50) - long-term endothelial cell survival and proliferation [37,38], the same experiment was performed on DS and euploid progenitors infected with a low MOI (about 50) of Bartonella. No differences in LP were observed in both DS and euploid infected cells compared to uninfected, in the absence of hydroperoxide treatment (data not shown). More interestingly, a significant decrease in LP was observed in Bartonella-infected DS progenitors vs uninfected (p < 0.01) (Figure 3A lower panel). This finding is in accordance to the previously reported beneficial effect of infection at low MOI of *B. henselae *[37,38].

Moreover, it is known that a constitutive increase in *S100B*, due to HSA21 trisomy, is likely to induce ROS generation, leading to increased oxidative stress in DS [39], and the over-expression of *SOD1 *gene has been suggested to be responsible of oxidative damage to neurons [40]. Thus, we measured the expression levels of both *S100B *and *SOD1 *genes in DS and euploid isolated EPCs, showing their significant over-expression in DS derived cells (Figure 3B).

### Chromosome 21 expression profile

We chose a user fold-change of 2 and a P-value cut-off of 0.005 for selecting a list of differentially expressed genes, and we first focused on the HSA21 genes according to GenBank annotation. Thus, to evaluate the impact of an extra copy of chromosome 21 on DS progenitors we performed a detailed analysis of HSA21 genes in DS vs eupolid EPCs. We observed that only 109 out of a total of 386 genes annotated on HSA21 (NCBI RefSeq 36.3), were detected in this microarray analysis. Furthermore, 52 (about 14% of total HSA21 annotated genes) showed an evidence of differential expression in DS EPCs compared to euploid cells. In particular, 37 genes were up- and 15 down-regulated (72% up- and 28% down-regulated, respectively) [Additional file 4: Supplemental Table S2].

**Figure 4:**
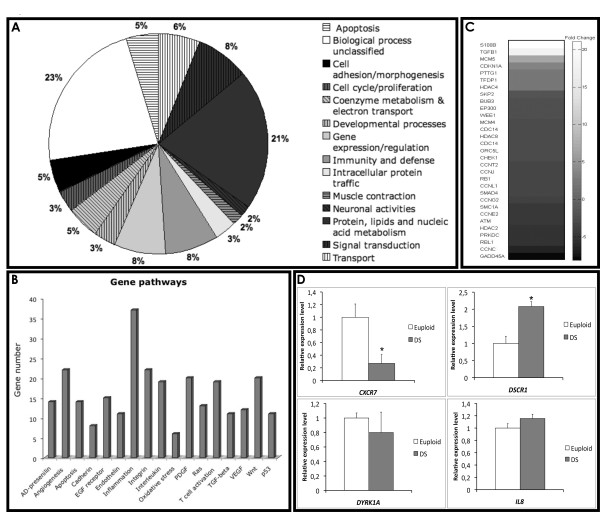
**Differentially expressed genes in DS vs euploid progenitors**. A) Schematic representation of chromosome 21 differentially expressed genes in DS vs euploid progenitors. Genes are categorized according to GO classification or their hypothetical biological function. B) Bar graph representation of more pronounced deregulated gene pathways in the whole genome of DS vs euploids, after PANTHER analysis. Vertical bars indicate the number of differentially expressed genes per pathway. C) HeatMap showing the fold-change of cell cycle and cell cycle-related differentially expressed genes in DS vs euploid progenitors. Gray intensity is proportional to the fold-change; the bar on the right illustrates the association between fold-changes and grayscale. The genes are ranked from the most up-regulated (white) to the most down-regulated (black). D) qRT-PCR for crucial genes belonging to the most deregulated gene pathways are shown. Data are indicated as relative expression levels (Euploid EPCs = 1).

Database searches based on GO classification, revealed that differentially expressed genes were mostly associated to immune response (GO:0006955) and transcription regulation (GO:0045449) (Figure 4A). Particularly, crucial genes involved in the immune response, such as interferon receptors (*IFNAR1 *and *IFNAR2*), and oxidative stress, such as SOD1, S100B and APP - recently implicated in DS neurotoxicity from elevated expression of free radicals [39] - were highly up-regulated in DS vs euploid EPCs (Figure 3B; Additional file 4: Supplemental Table S2).

Although we observed relatively small up-regulation of chromosome 21 genes, significant changes in gene expression were not limited to HSA21 genes. Indeed, we observed a global and pronounced deregulation overall the chromosomes, possibly explained by dosage imbalance of HSA21 genes encoding transcriptional factors or gene expression modulators (Figure 4A; Additional file 4: Supplemental Table S2).

A less frequently explored gene characteristic for microarray analysis is the chromosomal location of the genes, especially when studying diseases caused by genome alterations. First we demonstrated, by using chi-squared association tests, that differentially expressed genes are not uniformly distributed along the chromosomes. We observed that in DS EPCs the chromosomes 21 - as expected - and 19 were enriched for differentially expressed genes compared to the other chromosomes (α 0.001; *P *= 6.7E-0.6; Additional file 5: Supplemental Figure S2). As a proof of the robustness of our findings, the analysis was repeated with different fold-change and *P *cut-offs, reporting similar qualitative findings. Moreover, by using positional gene enrichment (PGE) approach [41] to map a set of genes to the exact location on chromosome [Additional file 6: Supplemental Figure S3], we observed that chromosome 19 has the highest percent of deregulated genes [Additional file 3: Supplemental Figure S1A] and, in particular, that the enriched p12 band contains most of the genes encoding for transcription factors of the zinc finger protein superfamily.

### Gene pathways' perturbation in DS progenitors

By using the same selection parameters, the comparison of global DS and euploid expression profiles revealed that, after filtering ("Materials and Methods"), 2913 genes (2489 of them with single probe and 424 with multiple probes) were differentially expressed, on a total of 11327 distinct genes considered. By using DAVID and PANTHER Classification System, differentially expressed genes were categorized according to their known or hypothetical biological function, and the enrichment for specific gene pathways was evaluated. The analysis revealed that most of the deregulated genes were involved in "angiogenesis", "inflammation mediated by cytokines and chemokines", "integrins" and "interleukines" signaling pathways (Figure 4B). A particular enrichment was also observed for cell cycle and cell cycle-related genes (Figure 4C).

Interestingly, angiogenesis inhibitors encoding genes - such as *CXCL10 *and other interferon-stimulated genes - were up-regulated in DS-derived progenitors, whereas, on the opposite, pro-angiogenic genes (*VEGFA*, *CXCL12*, *EDN1, CASP8*) were dramatically down-regulated (Table 1).

**Table 1:** Differential expression of angiogenesis-related genes in DS vs C endothelial progenitors

*Gene name*	*Gene symbol*	*RefSeq*	*Fold change*	*Function*
Chemokine (C-X-C motif) ligand 10	*CXCL10*	NM_001565	6.7 I	Antiangiogenic chemokine

Interferon stimulated gene 20	*ISG20*	NM_002201	4.4 I	Angiogenesis inhibitor

SAM domain- and HD domain-containing protein 1	*SAMHD1*	NM_015474	3.0 I	Interferon-γ stimulated; angiogenesis inhibitor

Caspase 8	*CASP8*	NM_001228	3.5 D	Adhesion and homing of EPC

Chemokine (C-X-C motif) ligand 12	*CXCL12*	NM_199168	10.2 D; 5.7 D	Mobilization and recruitment of EPCs

Chemokine (C-X-C motif) receptor 4	*CXCR4*	NM_001008540	3.0 D	Endothelial cells migration and homing

Chemokine (C-X-C motif) receptor 7	*CXCR7*	NM_020311	4.3 D	Endothelial cells migration and homing

Endothelin 1	*EDN1*	NM_001955	13.6 D; 3.5 D	Promotes migration and proliferation of endothelial cells

Endothelin receptor type B	*EDNRB*	NM_000115	2.8 D	Migration, proliferation of endothelial cells

Vascular endothelial growth factor A	*VEGFA*	NM_001025367	3.6 D	Angioproliferative

Stanniocalcin 1	*STC1*	NM_003155	56.0 D	VEGF-mediated angiogenic response

Since evidences indicate that chemokines, cytokines and soluble factors affect the mobilization and recruitment of endothelial progenitors [42,43], we evaluated the expression of some crucial genes by quantitative RT-PCR (Figure 1E and 4D). Particularly, we observed a down-regulation of *CXCR4 *receptor and of its ligand, encoded by *CXCL12 *gene, in DS progenitors compared to euploid cells (Figure 1E), the over-expression of *RCAN1 *gene (or *DSCR1*) and the down-regulation of *CXCR7 *receptor (Figure 4D), which play a key role in endothelial cells migration and homing. No significant differential expression was observed for *IL8 *and *DYRK1A *genes in DS progenitors compared to euploid (Figure 4D).

### *B. henseale*-induced gene expression variations

A similar approach for gene list selection was used to investigate the genetic response of DS-EPCs to *B. henselae *infection at a 100 MOI.

GO term enrichment analysis [38] showed that a considerable number of induced/repressed genes belong to immune and inflammatory response pathways [Additional file 7: Supplemental Figure S4], with the majority of genes annotated within the "Jak/STAT" and "Cytokine-cytokine receptor interaction" pathways. Furthermore, we categorized the most prominently up-regulated genes in DS infected EPCs in two related functional classes: "Interferons related/induced genes" and "cytokines and chemokines", consisting of 19 and 32 genes, respectively. The same approach was then used for infected euploid cells. Transcriptional levels of related genes, observed by microarray, are shown in [Additional file 8: Supplemental Table S3].

**Figure 5:**
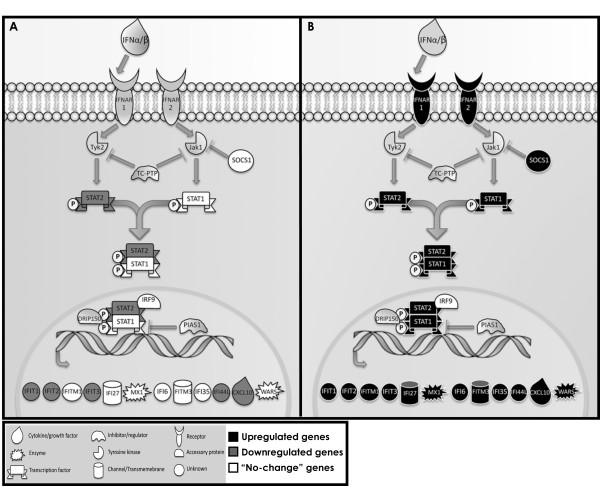
**JAK/STAT pathway in infected EPCs**. Graphical representation of interferon signalling pathway comprising many of the differentially expressed genes in infected DS vs euploid EPCs. JAK/STAT pathway up-, down-regulated and "no-change" genes are shown in black, gray and white, respectively.

We focused our interest toward the cluster of differentially expressed genes of the Jak/STAT pathway (Figure 5). The analysis revealed that DS infected EPCs have a very distinct "molecular signature" compared to infected euploid progenitors, mostly characterized by the up-regulation of interferon-stimulated genes (ISGs). Particularly, a large subset of differentially expressed crucial genes was also confirmed by semi-quantitative RT-PCR [Additional file 7: Supplemental Figure S4]. As a reflection of the robust induction of ISGs, 'IFN signaling' was identified as the top scored pathway induced in DS progenitors after infection of *B. henselae*.

Other deregulated genes - involved in cell cycle regulation and gene expression - were further identified, many of which are known to be targets of interferon action as a consequence of the anti-proliferative effects.

## Discussion

Alteration of EPC number has been described in a wide range of conditions, such as cardiovascular, inflammatory, immune, and infectious diseases [21,44]. A decreased *in vitro *growth capacity of bone marrow-derived progenitors in DS mouse model Ts65Dn [24] and a reduced number of CD34+ in DS fetuses and children were also reported [22]. More recently, Diller et al. (2008) reported an impairment of CD34+/AC133+/KDR+ cells in a small subset of DS individuals affected by Eisenmenger syndrome [23]. Despite the use of different experimental approaches and the limited number of individuals, the reduced number of circulating progenitors is an important common finding. These evidences may possibly account for the differences in angiogenesis, inflammatory and immune response reported in DS [14].

Here, we have shown that DS patients exhibit a marked reduction of ≈ 40% in the number of EPCs, also displaying a significant increase in cell size and major detrimental morphological changes (i.e., cell vacuolization and high number of active lysosomes). The reduced number of progenitors could be associated with alterations in the cell cycle; however, we did not find any significant difference between the groups [Additional file 3: Supplemental Figure S1C]. Thus, we investigated other possible mechanisms responsible for the observed EPCs impairment, such as mobilization/homing and oxidative stress susceptibility.

Growing evidences indicate that chemokines and cytokines, such as SDF-1α and its receptor CXCR4, play a crucial role in the mobilization and homing of EPCs from bone marrow [45,46], also affecting cell proliferation [41,42]. Our findings of a significant decrease in SDF-1α plasma levels in young DS - compared to age-matched euploids - and a strong decrease of *CXCL12 *and *CXCR4 *gene transcription in their EPCs, suggest a link with the reduced number of circulating progenitors and the angiogenesis suppression observed in DS.

These results are strengthened by microarray analysis, indicating that DS progenitors have a pronounced perturbation, at least at transcriptional level, in the angiogenesis and cell cycle pathways. Indeed, by using this approach we demonstrated the transcriptional deregulation of *CXCR7*, *IL8 *and *RCAN1 *genes, crucial factors involved in endothelial cells' migration, homing and angiogenesis [47-49]. Moreover, we found a down-regulation of *CASP8*, which has been demonstrated to have a novel apoptosis-unrelated role in proangiogenic cells [50], although this gene was found to be associated with breast cancer by gene-based association study, and its down-regulation has also been reported in breast cancer [51].

We recently demonstrated the relevance of oxidative stress on the number and function of progenitor cells [52]. Besides, it is known that oxidative stress is a crucial issue in the pathogenesis of DS, especially due to the high incidence of Alzheimer-like disease at young age [53]. It has been also suggested that constitutive expression of trisomic genes, *S100B *and *SOD1*, is likely to represent a leading cause of ROS generation and increased oxidation in DS neurons [39,40]. Oxidative stress relevance in DS fetuses was also highlighted by microarray analysis of uncultured amniotic fluid [54].

Here, we have assessed, by both experimental evidences and microarray analysis, that DS progenitors are more susceptible to hydroperoxide-induced LP and significantly over-express *S100B *and *SOD1 *genes. These findings strengthen the hypothesis of their involvement in the susceptibility to oxidation observed in DS endothelial progenitors.

In our study we have infected DS progenitors with a human pathogen, *B. henselae*, responsible of vasoproliferative diseases such as bacillary angiomatosis and peliosis in immunocompromised patients [27,28], previously demonstrated to adhere to and invade EPCs [26]. Particularly, we investigated the effect of infection on DS progenitors' number, morphology and oxidative stress response. After hydroperoxide treatment, we observed a significant LP decrease in Bartonella-infected DS progenitors compared to uninfected DS cells. This finding confirms the beneficial effect of *B. henselae *infection at low MOI on mature endothelial cells' survival [37,38].

In contrast, a reduced cell number in both DS and euploid groups was observed after Bartonella infection at higher MOIs; however, detrimental changes were visible only in DS EPCs, displaying a higher number of invasomes and infected cells compared to euploid cells.

The molecular basis of such Bartonella-induced detrimental effect on endothelial progenitors of DS was investigated at a transcriptional level by microarray. Significant up-regulation of the Jak/STAT pathway was observed only within infected DS progenitors, whereas, on the opposite, infected euploid cells displayed a significant down-regulation. These findings strengthen the hypothesis that transcriptional analysis of EPCs is clearly of major interest in the context of this syndrome. Indeed, the activation of ISGs, involved in the immune response against infections and in tumor surveillance [55], also inhibits angiogenesis by decreasing the production of angiogenic factors such as VEGF and IL8 [56]. Our results clearly show that a similar ISGs activation occurs in infected DS progenitor cells, and, albeit at lower levels, in uninfected DS progenitors (data not shown).

Pathologic immune and inflammatory responses are regulated by the cross-talk between interferons and TNFα [26], as well as deregulation of chemokines/cytokines greatly affects the mobilization and recruitment of endothelial progenitors. The imbalance between ISGs and other molecules might be of great immunological relevance concerning the well-known DS haematological defects.

## Conclusions

Physiological angiogenesis plays a central role in the embryogenesis and placental development; on the other hand, pathological angiogenesis represents a critical issue in the progression of many diseases, such as solid tumor growth and retinopathy.

Individuals with Down syndrome, due to decreased incidence of angiogenesis-dependent diseases, have been postulated to be a systemic anti-angiogenesis model. Indeed, they exhibit a significantly increased anti-angiogenic surveillance, possibly due to increased expression of anti-angiogenic regulators on chromosome 21 [57].

However, it has been shown a complex regulation of gene expression not only related to gene copy number, with several genes escaping the rule of "increased transcription proportional to the gene copy number" [58,59]. This findings suggest that many pathological traits observed in DS may be controlled by other more complex and, above all tissue-specific, regulatory mechanisms [59].

Our study shows that circulating endothelial progenitors are reduced in patients with DS, possibly correlating to the low SDF-1α plasma levels, to a reduced expression of its membrane receptor in these cells, and to their higher oxidative stress and pathogen infection susceptibility compared to euploid cells. A significant perturbation in the angiogenesis and inflammation gene pathways was also observed by microarray analysis, highlighting that gene expression analysis is a crucial issue for the study of common diseases. Endothelial dysfunction, angiogenesis' suppression and infection recurrence are hallmarks of DS, and the impairment in the number and function of circulating progenitors may account for some of their pathological features. Further studies are needed to understand possible therapeutic implications of circulating EPCs in Down syndrome.

## Competing interests

The authors declare that they have no competing interests.

## Authors' contributions

CV, LS, ACa, BA, PS, ACi and CN designed research. CV, LS, ACa, RCo, VM, LM, CF, MR and MP carried out experimental research. VM and BA performed Transmission electron microscopy. CV and MR performed microarray analysis and quantitative RT-PCR. CV and CAn assisted with statistical analysis. BF, MMC, BS and RCa participated in the participant recruitment. CV, LS, ACa, RCo, CAn, VM, LM, BF, AG, CF, MR, MP, BA, MMC, BS, RCa, PS, ACi and CN analyzed data. CV, LS, ACa, CAn, BA, PS, ACi and CN wrote the paper. All authors read and approved the final manuscript.

## Pre-publication history

The pre-publication history for this paper can be accessed here:

http://www.biomedcentral.com/1755-8794/3/40/prepub

## Supplementary Material

Additional file 1 Supplementary Methods Click here for file

Additional file 2 Table S1: Primer pairs used for quantitative and semi-quantitative RT-PCR Click here for file

Additional file 3 **Figure S1: Impaired EPC number and function**. A) Representative photomicrographs of merged double-positive Dil-Ac-LDL/Lectin cells isolated from euploid (left panel) and DS (right panel) subjects (100X magnification). B) Fluorescence micrographs of EPCs labeled for 30 min with C11-BO in euploid and DS subjects. C) EPC number expressed as percentage in the different phases of cell cycle obtained by FACS. D) Curves indicate the percentage of EPC number infected with *B. henselae *in euploid and DS individuals. Results are representative of five different experiments in duplicate. Click here for file

Additional file 4 Table S2: Chromosome 21 genes differentially expressed in DS vs euploids Click here for file

Additional file 5 **Figure S2: Distribution of differentially expressed genes along the human chromosomes (DS vs euploids)**. A) Bar graph showing the empirical frequency distribution of differentially expressed genes along the autosomes of DS progenitors vs euploids. Asterisks indicate the significantly deregulated chromosomes. B) Representation of the robustness of our findings shown in A. The left column shows the different user-defined fold-change. For each á value used in the analysis are shown the relative p-values. C) Bar graph showing the percent of differentially expressed genes along the DS autosomes. Click here for file

Additional file 6 **Figure S3: Positional gene mapping of differentially expressed genes (DS vs euploids)**. Graphic representation of positional gene enrichment (PGE) approach used to map differentially expressed genes in DS vs euploids EPCs to the exact location on the chromosome. Click here for file

Additional file 7 **Figure S4: *B. henseale*-induced gene expression in DS EPCs**. A) Bar graph showing the top-scored deregulated gene pathways after infection in DS progenitors. Ratio indicates the percent of differentially expressed genes within the related pathway. B) Semiquantitative RT-PCR of Jak/STAT genes deregulated after *B. henseale *infection. Click here for file

Additional file 8 Table S3: Differentially expressed genes after *B. henselae *infection Click here for file
