# Supplementary material for: Impairment of circulating endothelial progenitors in Down syndrome
Source: BMC Med Genomics. 2010 Sep 13;3:40. doi: 10.1186/1755-8794-3-40 (PMC2949777; doi:10.1186/1755-8794-3-40)
Supplement: Additional file 1 — Supplementary Methods [file 1755-8794-3-40-S1.DOC]

**Supplementary of “Microarray analysis and quantitative RT-PCR”**

Total RNA (10 μg) was isolated from young age-matched DS and euploid individuals' cultured EPCs, infected with Bartonella at MOI of 100, and uninfected, by RNAzol B (Campro Scientific) according to the manufacturer’s instructions^31^. After treatment with DNase I (Gibco BRL), RNA was used for reverse transcription with random hexamers and Superscript III (Gibco BRL)^31^.

We observed strong reproducibility between the euploid arrays, then we averaged intensities. After filtering out probes with a signal intensity <50 in DS and mean-euploid arrays, and/or <10 in one condition, we obtained 19826 probes, corresponding to 11327 genes according to Affymetrix annotation (6314 genes with single probe and 5013 genes with multiple). Distinct genes were classified up-, down-regulated and “no-change”, and differentially expressed genes were selected for further analysis. Quantitative and semi-quantitative RT-PCRs were performed as described^31^. Primer pairs of selected genes are listed in Additional file 2: Supplemental Table S1.

**Supplementary of “*In silico* significant pathway identification”**

Following the suggestions of DAVID’s creators^32,33^, we examined functional annotations with a Fisher exact *p*-value (‘‘EASE’’ score) <0.1. We used the “Functional annotation clustering” option to cluster functionally similar terms of user’s gene list into groups. This option allows to explore it group by group rather than single terms one by one, ranking the importance of annotation groups with the enrichment score parameter^33^.

**Supplementary of “Statistical analysis”**

EPCs number and cell size were evaluated at least in 6 different DS and 20 euploid donors, repeated three times for each individual, revealing comparable results. Two independent observers examined samples blindly to cell type.

Oxidation was evaluated by C11-BO and performed in two separate experiments on six DS and euploid age-matched individuals. Fluorescence was measured at least in three different fields for each sample per condition.

For microarray analysis, single probes were directly classified up-, down-regulated and “no-change” according to a user-defined fold-change. Multiple probes underwent a consistency analysis to assign them to the above-mentioned classes. First, we identified genes with multiple probes showing consistent fold-change assignment (i.e., probes with fold-change above or below the user-defined threshold), and those with inconsistent behaviour (two probes above the threshold with different signs). The former were classified, and the latter, for whose annotation was not clear, were removed. Then, a z-test with pooled variance (median of the genes' sample variance) was used to further discriminate consistent probes from questionable signals. A gene was considered differentially expressed if the null hypothesis of “no-change” was rejected according to a cut-off *p*-value (at least 0.05), *vice-versa* the gene was classified as “no-change”. The classification was repeated with different user-defined fold-changes and different cut-off *p*-values to balance the false positive and negative errors.

**Figure S1: Impaired EPC number and function**

**A)** Representative photomicrographs of merged double-positive Dil-Ac-LDL/Lectin cells isolated from euploid (left panel) and DS (right panel) subjects (100X magnification). **B)** Fluorescence micrographs of EPCs labeled for 30 min with C11-BO in euploid and DS subjects. **C)** EPC number expressed as percentage in the different phases of cell cycle obtained by FACS. **D)** Curves indicate the percentage of EPC number infected with *B. henselae* in euploid and DS individuals. Results are representative of five different experiments in duplicate.

**Figure S2: Distribution of differentially expressed genes along the human chromosomes (DS vs euploids)**

**A)** Bar graph showing the empirical frequency distribution of differentially expressed genes along the autosomes of DS progenitors vs euploids. Asterisks indicate the significantly deregulated chromosomes. **B)** Representation of the robustness of our findings shown in A. The left column shows the different user-defined fold-change. For each α value used in the analysis are shown the relative p-values. **C)** Bar graph showing the percent of differentially expressed genes along the DS autosomes.

**Figure S3: Positional gene mapping of differentially expressed genes (DS *vs* euploids)**

Graphic representation of positional gene enrichment (PGE) approach used to map differentially expressed genes in DS vs euploids EPCs to the exact location on the chromosome. Blue, green and brown bars indicate the number of “no change”, upregulated and downregulated genes, respectively.

**Figure S4: *B. henseale*-induced gene expression in DS EPCs**

**A)** Bar graph showing the top-scored deregulated gene pathways after infection in DS progenitors. Ratio indicates the percent of differentially expressed genes within the related pathway. **B)** Semiquantitative RT-PCR of

Jak/STAT genes deregulated after *B. henseale* infection.
