# Supplementary material for: Impairment of circulating endothelial progenitors in Down syndrome
Source: BMC Med Genomics. 2010 Sep 13;3:40. doi: 10.1186/1755-8794-3-40 (PMC2949777; doi:10.1186/1755-8794-3-40)
Supplement: Additional file 2 — Table S1: Primer pairs used for quantitative and semi-quantitative RT-PCR [file 1755-8794-3-40-S2.DOC]

**Table S1. Primer pairs used for quantitative and semi-quantitative RT-PCR**

| **Gene symbol** | **Primer sequence (5’-3’)** | |
| --- | --- | --- |
|  | ***Forward*** | ***Reverse*** |
| *CXCR7* | TTTCATCTTCGTCATCGGCA | AGATGAGGTGTGTGACTTTG |
| *CXCL12* | AAGGTCGTGGTCGTGCTGG | GGGCTACAATCTGAAGGGGCA |
| *CXCR4* | CAGATAACTACACCGAGGAAA | AGCGTGATGACAAAGAGGAG |
| *IL8* | TGCCAAGGAGTGCTAAAGAA | CAAAAACTTCTCCACAACCC |
| *RCAN1* | TTGACTGCGAGATGGAGGA | GAAGGGGTTGCTGAAGTTTA |
| *DYRK1* | CCTTGCGACTCCAGAACTT | TCAACCAAAATACACCCGAG |
| *SOD1* | AGTGAAGGTGTGGGGAAGC | CAATGATGCAATGGTCTCCT |
| *S100B* | CATCGACGTTTTCCACCAATA | TCGTGGCAGCGAGTAGTAAC |
|  |  |  |
| *IFNAR1* | GGAACAGGAGCGATGAGTCT | CACTATTGCCTTATCTTCAGC |
| *IFNAR2* | ACCACCAGAGTTTGAGATTGT | GGAGACTTTATTACTGCTTGC |
| *JAK1* | CCTCTTTGCCCTGTATGACG | CATACTGTCCCTGAGCAAAC |
| *STAT1* | AAAAGCAAGACTGGGAGCAC | ATTCCCCGACTGAGCCTGAT |
| *STAT2* | GAGAAAGGAGGTGCTGGATG | TGACCTGGGCGTTGCGTAG |
| *IFIT3* | TGCAGGGAAACAGCCATCAT | ACTTCTGATTTCTGCTTGGTC |
| *IFITM1* | TGTCGTCTGGTCCCTGTTC | CCCGTTTTTCCTGTATTATC |
