# Supplementary figures and images for: Impairment of circulating endothelial progenitors in Down syndrome
Source: BMC Med Genomics. 2010 Sep 13;3:40. doi: 10.1186/1755-8794-3-40 (PMC2949777; doi:10.1186/1755-8794-3-40)

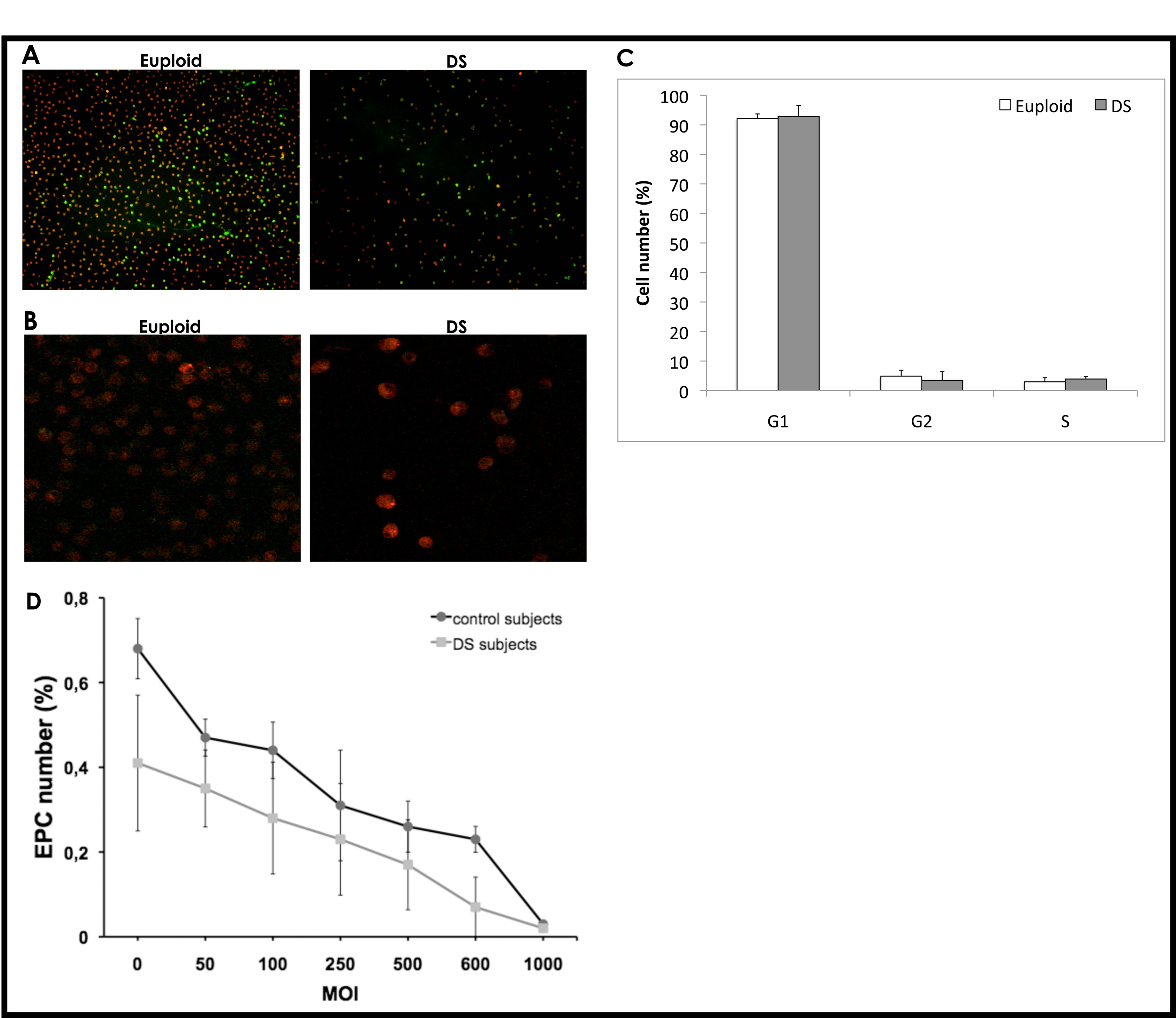

Supplement: Additional file 3 — Figure S1: Impaired EPC number and function. A) Representative photomicrographs of merged double-positive Dil-Ac-LDL/Lectin cells isolated from euploid (left panel) and DS (right panel) subjects (100X magnification). B) Fluorescence micrographs of EPCs labeled for 30 min with C11-BO in euploid and DS subjects. C) EPC number expressed as percentage in the different phases of cell cycle obtained by FACS. D) Curves indicate the percentage of EPC number infected with B. henselae in euploid and DS individuals. Results are representative of five different experiments in duplicate. [file 1755-8794-3-40-S3.JPEG]

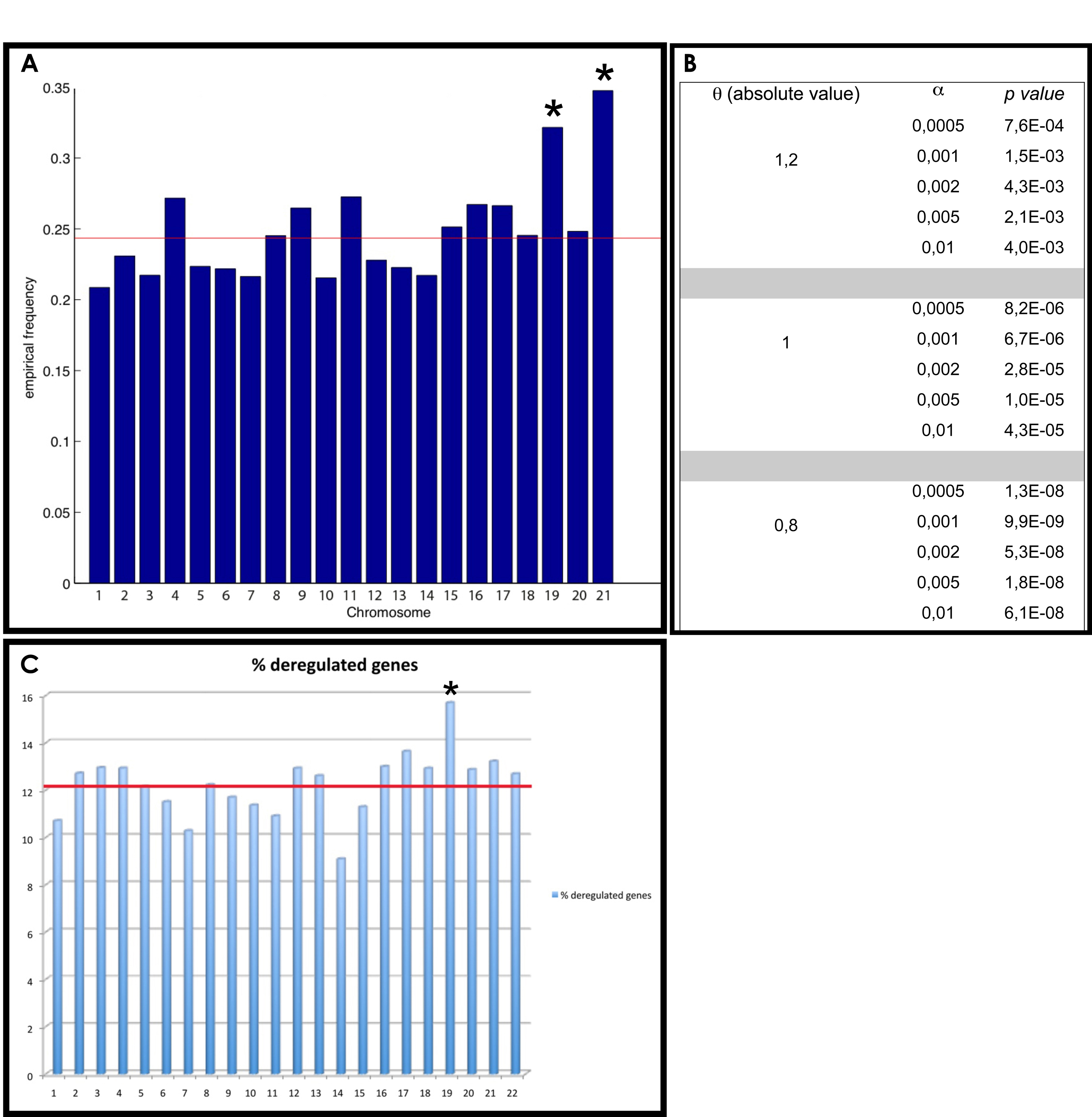

Supplement: Additional file 5 — Figure S2: Distribution of differentially expressed genes along the human chromosomes (DS vs euploids). A) Bar graph showing the empirical frequency distribution of differentially expressed genes along the autosomes of DS progenitors vs euploids. Asterisks indicate the significantly deregulated chromosomes. B) Representation of the robustness of our findings shown in A. The left column shows the different user-defined fold-change. For each á value used in the analysis are shown the relative p-values. C) Bar graph showing the percent of differentially expressed genes along the DS autosomes. [file 1755-8794-3-40-S5.JPEG]

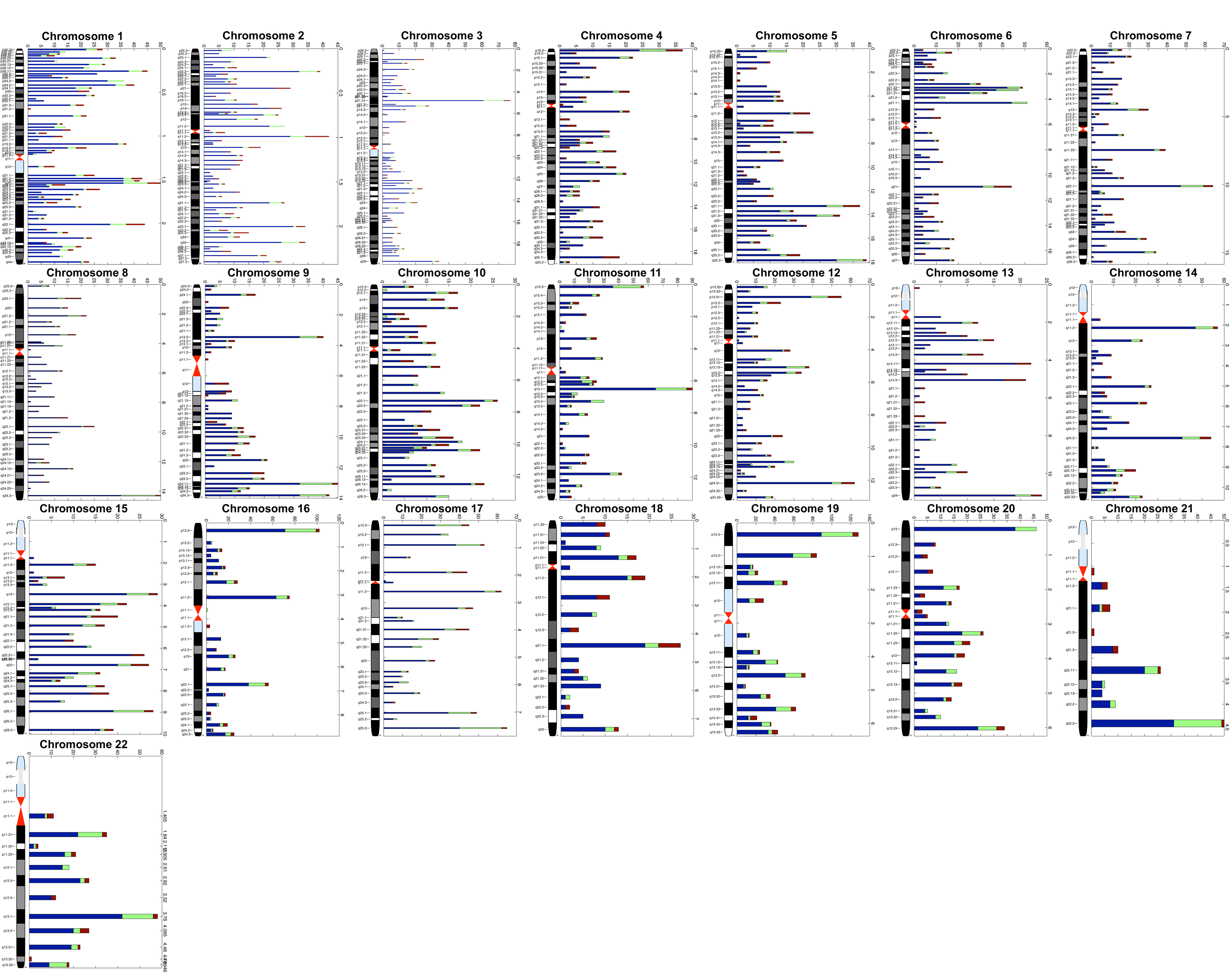

Supplement: Additional file 6 — Figure S3: Positional gene mapping of differentially expressed genes (DS vs euploids). Graphic representation of positional gene enrichment (PGE) approach used to map differentially expressed genes in DS vs euploids EPCs to the exact location on the chromosome. [file 1755-8794-3-40-S6.JPEG]

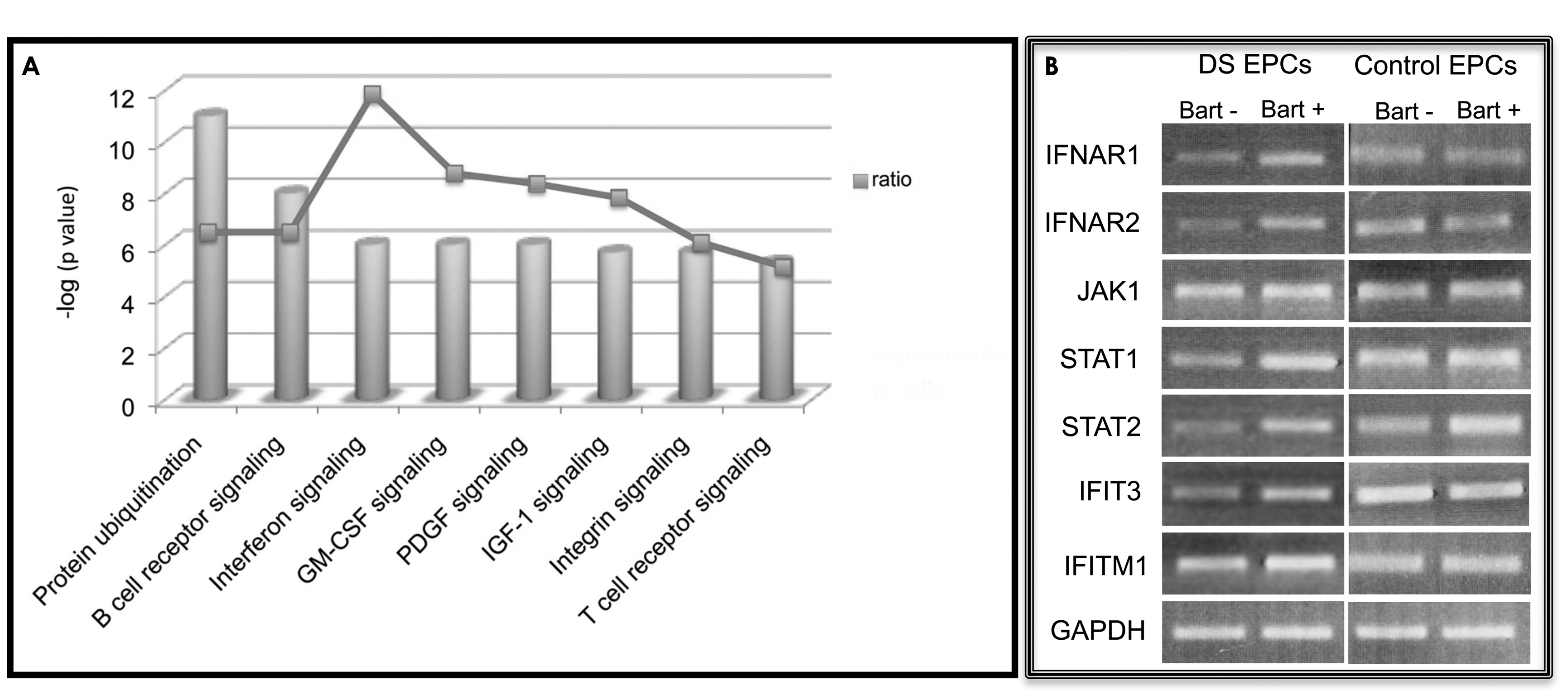

Supplement: Additional file 7 — Figure S4: B. henseale-induced gene expression in DS EPCs. A) Bar graph showing the top-scored deregulated gene pathways after infection in DS progenitors. Ratio indicates the percent of differentially expressed genes within the related pathway. B) Semiquantitative RT-PCR of Jak/STAT genes deregulated after B. henseale infection. [file 1755-8794-3-40-S7.JPEG]
