# Supplementary material for: Impairment of circulating endothelial progenitors in Down syndrome
Source: BMC Med Genomics. 2010 Sep 13;3:40. doi: 10.1186/1755-8794-3-40 (PMC2949777; doi:10.1186/1755-8794-3-40)
Supplement: Additional file 4 — Table S2: Chromosome 21 genes differentially expressed in DS vs euploids [file 1755-8794-3-40-S4.DOC]

**Table S2.** Chromosome 21 genes differentially expressed in DS vs euploids

| **Gene symbol** | **Functional assignment** | **DS *vs.* C** |
| --- | --- | --- |
| **Immune response and immune system** | | |
| *CCT8* | T-complex subunit | 2.5 I |
| *CSTB* | Associated to progressive myoclonic epilepsy | 2.0 I |
| *ICOSLG* | Co-stimulatory signal for T-cell proliferation and cytokine secretion | 2.8 I |
| *IFNAR1* | Receptor/auxilliary factor | 4.8 I |
| *IFNAR2* | Receptor/auxilliary factor | 3.4 I |
| *ITGB2* | Integrin protein | 7.8 I |
| *SOD1* | Antioxidant activity | 2.2 I |
| *TIAM1* | T-lymphoma invasion & metastasis inducing protein | 3.1 I |
| *ADAMTS5* | Cleaves aggrecan, a major proteoglycan of cartilage | 3.3 D |
| *BAGE* | B melanoma antigen | 9.2 D |
| *CXADR* | Coxsackievirus and adenovirus receptor precursor | 8.5 D |
| *JAM2* | lymphocyte homing to lymphoid organs | 5.6 D |
| **Transcription factors and gene expression/modulation** | | |
| *ADARB1* | Pre-mRNA editing of the glutamate receptor subunit B | 6.1 I |
| *BRWD1* | Putative transcriptional activator; chromatin remodeling | 2.2 I |
| *DONSON* | Downstream of *SON*; function unknown | 2.1 I |
| *PRDM15* | Transcriptional regulation | 2.6 I |
| *PRMT2* | Transcriptional coactivation | 3.7 I |
| *RUNX1* | Transcription factor | 2.5 I |
| *SAMSN1* | SH3 domain and nuclear localization signals protein | 2.1 I |
| *SON* | DNA binding domain | 2.6 I |
| *TTC3* | Zinc finger, RING-type | 2.1 I |
| *ZNF294* | Zinc finger | 2.0 I |
| *ZNF295* | Zinc finger | 2.1 I |
| *WDR4* | Gene regulation; apoptosis | 3.0 I |
| *ETS2* | TF involved in SC development, ageing and death | 3.9 D |
| *GABPA* | Transcription factor | 2.1 D |
| *NRIP1* | Modulator of transcriptional activation by estrogen | 2.5 D |
| *SFRS15* | Splicing factor | 2.1 D |
| *U2AF1* | Splicing factor | 2.0 D |
| **Cell morphogenesis/cell size** | | |
| *ITGB2* | Integrin protein | 7.8 I |
| *PCNT* | Functioning of centrosomes; cell-cycle progression | 3.1 I |
| *S100B* | Regulation of cell cycle progression | 21.2 I |
| **Cellular transport (vescicle-mediated, channels)** | | |
| *ATP5J* | ATP synthase, H+ transporting | 2.0 I |
| *DSCR3* | Contributes to pathogenesis of DS (vacuolar transport) | 2.1 I |
| *KCNE1* | Delayed rectifier potassium channel | 2.6 I |
| *PTTG1IP* | Facilitates PTTG1 nuclear translocation potentiating its activation | 2.7 I |
| *SLC19A1* | Folate transporter | 3.5 I |
| *TRPM2* | Transient receptor potential cation channel | 3.8 I |
| *ABCC13* | ATP-binding cassette protein | 4.4 D |
| *ABCG1* | ATP-binding cassette involved in cholesterol and PL transport | 5.9 D |
| *DOPEY2* | Golgi to endosome transport | 3.3 D |
| *SLC5A3* | Solute carrier family 5 (inositol transporter) | 4.8 D |
| **Cellular metabolic activity and protein degradation** | | |
| *AGPAT3* | De novo phospholipid biosynthetic pathway | 2.5 I |
| *PDXK* | Phosphorylates vitamin B6 | 2.7 I |
| *PIGP* | GPI-anchor biosynthesis | 2.1 I |
| *POFUT2* | O-fucosyltransferase | 2.4 I |
| *PSMG1* | Proteasome assembly chaperone 1 | 2.8 I |
| *SUMO3* | Protein sumoylation | 3.3 I |
| *UBASH3A* | Ubiquitin-conjugating enzyme | 3.0 I |
| *UBE2G2* | Ubiquitin-conjugating enzyme | 4.4 I |
| *USP16* | Deubiquitinating enzyme involved in mitosis | 2.9 I |

I = increase; D = decrease.
