# Supplementary material for: Impairment of circulating endothelial progenitors in Down syndrome
Source: BMC Med Genomics. 2010 Sep 13;3:40. doi: 10.1186/1755-8794-3-40 (PMC2949777; doi:10.1186/1755-8794-3-40)
Supplement: Additional file 8 — Table S3: Differentially expressed genes after B. henselae infection [file 1755-8794-3-40-S8.DOC]

**Table S3. Differentially expressed genes after *B.henselae*** infection

| ***Gene symbol*** | ***Gene name*** | ***Infected DS*** | ***Infected C*** |
| --- | --- | --- | --- |
| *IFN related/induced genes* | | | |
| *CXCL10* | chemokine (C-X-C motif) ligand 10 | 5.4 I | 2.2 D |
| *GBP1* | guanylate binding protein 1. interferon-inducible. 67kDa | 3.0 I | 2.1 D |
| *IFI27* | interferon. alpha-inducible protein 27 | 5.1 I | N.C. |
| *IFI44L* | interferon-induced protein 44-like | 3.3 I | 3.6 D |
| *IFI6* | interferon. alpha-inducible protein 6 | 2.7 I | N.C. |
| *IFIT1* | interferon-induced protein with tetratricopeptide repeats 1 | 3.0 I | 2.7 D |
| *IFIT2* | interferon-induced protein with tetratricopeptide repeats 2 | 2.9 I | 4.0 D |
| *IFIT3* | interferon-induced protein with tetratricopeptide repeats 3 | 4.6 I | N.C. |
| *IFITM3* | interferon induced transmembrane protein 3 | 2.0 I | N.C. |
| *IFRD1* | interferon-related developmental regulator 1 | 2.2 I | N.C. |
| *IKBKE* | inhibitor of kappa light polypeptide gene enhancer in B-cells | 2.4 I | N.C. |
| *INDO* | indoleamine-pyrrole 2.3 dioxygenase | 78.6 I | N.C. |
| *INDOL1* | indoleamine-pyrrole 2.3 dioxygenase-like 1 | 17.7 I | N.C. |
| *IRF1* | interferon regulatory factor 1 | 2.0 I | N.C. |
| *MX1* | myxovirus (influenza virus) resistance 1 | 2.0 I | N.C. |
| *PSMA8* | proteasome (prosome. macropain) subunit. alpha type. 8 | 2.2 I | 2.1 D |
| *PSMB2* | proteasome (prosome. macropain) subunit. beta type. 2 | 2.1 I | 2.0 D |
| *STAT1* | signal transducer and activator of transcription 1. 91kDa | 2.0 I | N.C. |
| *WARS* | tryptophanyl-tRNA synthetase | 5.2 I | N.C. |
| *Cytokines & Chemokines* | | | |
| *CCL13* | chemokine (C-C motif) ligand 13 | 2.7 I | 2.3 I |
| *CCL2* | chemokine (C-C motif) ligand 2 | 3.2 I | 2.3 I |
| *CCL20* | chemokine (C-C motif) ligand 20 | 3.9 I | 6.4 D |
| *CCL3* | chemokine (C-C motif) ligand 3 | 5.0 I | N.C. |
| *CCL4* | chemokine (C-C motif) ligand 4 | 2.6 I | N.C. |
| *CCL7* | chemokine (C-C motif) ligand 7 | 12.7 I | N.C. |
| *CCL8* | chemokine (C-C motif) ligand 8 | 9.1 I | 3.5 D |
| *CX3CR1* | chemokine (C-X3-C motif) receptor 1 | 2.4 I | N.C. |
| *CXCL1* | chemokine (C-X-C motif) ligand 1 | 9.6 I | N.C. |
| *CXCL16* | chemokine (C-X-C motif) ligand 16 | 2.0 I | N.C. |
| *CXCL2* | chemokine (C-X-C motif) ligand 2 | 2.3 I | N.C. |
| *CXCL5* | chemokine (C-X-C motif) ligand 5 | 6.4 I | N.C. |
| *CXCL9* | chemokine (C-X-C motif) ligand 9 | 11.8 I | N.C. |
| *FCAR* | Fc fragment of IgA. receptor for | 4.4 I | 3.3 D |
| *FCER1G* | Fc fragment of IgE. high affinity I. receptor for g polypeptide | 2.0 I | N.C. |
| *FCGR1A* | Fc fragment of IgG. high affinity Ia. receptor (CD64) | 3.7 I | 2.0 I |
| *FCGR1B* | Fc fragment of IgG. high affinity Ib. receptor (CD64) | 3.3 I | N.C. |
| *FCRL4* | Fc receptor-like 4 | 3.2 I | N.C. |
| *IL12RB1* | Interleukin 12 receptor. beta 1 | 4.3 I | N.C. |
| *IL17RA* | Interleukin 17 receptor A | 2.1 I | N.C. |
| *IL17RB* | Interleukin 17 receptor B | N.C. | 2.1 D |
| *IL1B* | interleukin 1. beta | 12.1 I | 2.9 D |
| *IL1R1* | interleukin 1 receptor. type I | 2.1 I | 2.2 I |
| *IL1R2* | interleukin 1 receptor. type II | 4.9 I | 3.7 I |
| *IL1RN* | interleukin 1 receptor antagonist | 7.8 I | N.C. |
| *IL2RA* | interleukin 2 receptor. alpha | 2.3 I | 4.2 I |
| *IL31RA* | interleukin 31 receptor A | 8.3 I | N.C. |
| *IL32* | interleukin 32 | 4.6 I | N.C. |
| *IL4I1* | interleukin 4 induced 1 | 2.9 I | N.C. |
| *IL8* | interleukin 8 | 7.7 I | N.C. |
| *IL9R* | interleukin 9 receptor | 2.0 I | N.C. |
| *TGFBR3* | transforming growth factor. beta receptor III | 4.3 I | N.C |
